# Exploring psychological well‐being in business and economics arena: A bibliometric analysis

**DOI:** 10.1002/hsr2.1044

**Published:** 2023-01-06

**Authors:** Satish Ambhore, Elvis K. Ofori

**Affiliations:** ^1^ Central Railway Headquarters, Office of Chief Safety Officer Chhatrapati Shivaji Maharaj Terminus Mumbai India; ^2^ School of Management Engineering Zhengzhou University Henan Zhengzhou China

**Keywords:** bibliometric, business, economics, job satisfaction, psychological well‐being

## Abstract

**Background:**

Recent events like the global pandemic and geopolitics leading to war bring to bear the evergreen importance of psychological well‐being (PWB) among workers and how it can further influence business growth and performance. Furthermore, the complexity of today's job requirements has created enormous life pressures for individuals, negatively hurting their PWB.

**Method:**

This article took the format of a literature review of existing research work by pursuing the keywords in the SCOPUS database to retrieve the articles published on PWB in the field of business and economics from 1978 to 2022. The data were analyzed to elaborate, interpret and graphically display the results, in particular, authors, sources, documents, and social structure of the existing bibliography. The Bibliometrix R package is used for robust analysis of retrieved data.

**Results:**

The findings showed that the last decade saw a rise in scholarly work on PWB. However, in 2021, its sharp expansion stalled. It further revealed that academics from four countries had a significant role in accessing PWB in the business and economics fields, namely the United States, the United Kingdom, Australia, and Canada. The reports also indicate themes such as mental health, coronavirus disease 2019 (COVID‐19), and depression are emerging themes, whereas niche themes include unemployment, quality of life, and job loss.

**Conclusion:**

This study suggests these new areas be studied in contemporary literature to provide cogent room to improve policy decisions on PWB within the business world.

## 1 INTRODUCTION

Psychological well‐being (PWB) connotes the extent to which individuals are thriving in their personal lives.^1^ It has piqued academic curiosity,^2^ but the coronavirus disease 2019 (COVID‐19) devastation and warning drew a strong spotlight on PWB in company development and performance.^3^ PWB is essential within this 21st century work environment because it provides a framework to handle intricacy within the everchanging work environment.^4^, ^5^ Furthermore, the complexity of today's job requirements has created enormous life pressures for individuals, negatively hurting their PWB.^6^ This provides context to inquire, "Does PWB have a relationship with work performance?" And even if it does, what is the nature of such a relationship? Indeed, several studies have demonstrated that employee satisfaction has a range of personal and organizational advantages, including improved firm productivity, but what about their PWB? What is less clear is how employee PWB influences work performance.^7^ According to research, employee health and well‐being are among the most important aspects influencing corporate success and performance.^8^ The second major gap is that various researchers understand PWB differently depending on their topic or study.^9^ Despite the increased academic interest in PWB, there are several awareness gaps in the extant literature. First, PWB's impact on business and economic development has not been widely debated.

The bibliometric review has been undertaken to foster an understanding of this situation best. Bibliometric analysis is a robust yet convenient method for examining the evolution of study domains, which is an integral part of assessing academic production. Importantly, additional study into the interaction between employees' PWB and work duties is required to progress job performance exploration.^10^ Therefore, this article tries to bring into perspective the need to have a retrospection of the period of scientific work and its impacts. This will provide context to identify gaps in academia and area of specialization quickly. The approach of this study was to conduct a bibliometric evaluation of the research on PWB, especially its impact on businesses and economies; this study synthesizes this scientific knowledge.

### 1.1 Background

The world, as everybody knows, is evolving rapidly. This has made daily activity very robust, and over the past decade, professional stress has become a significant worry worldwide. Despite rising knowledge of the effect of stress on corporate strategy, many businesses are still unable to tackle this problem effectively.^11^ According to global data, mental health issues are a factor in many people quitting their jobs^12^ and effect substantially influence how a company succeeds. Therefore, identifying mental health difficulties at work is a silver lining among all the chaos and pain. However, employers are beginning to recognize these issues in 2019 and the need to combat stigma and the increasing relationship between multiculturalism, equity, and integration. COVID‐19, through a pandemic, highlighted the old problem often overlooked in academia. This study will evaluate the research to best offer a path forward for understanding the phenomenon and potential policy suggestions for future work.

#### 1.1.1 The contemporary importance of PWB in business

Scholars believe there is a positive relationship between the economy and PWB as a sound man is efficient and adds to output.^13^, ^14^ Also, when the economy is good, and the standard of living is relatively high. Then, everyone (workers) enjoys the benefits and is excited. In furtherance of this analogy, the economic shock caused by COVID‐19 will significantly impact vulnerable groups' mental health, children, and workers who were highest with the phenomenon and well‐being during the downturns. However, businesses have been slow to respond. Regarding the economy, workplace mental health issues result in higher unemployment rates and lower productivity.^15^, ^16^ Most companies have yet to prioritize psychological state and well‐being because they are unaware of the economic advantages and lack information on the best ways to help their employees.

#### 1.1.2 COVID‐19: Impacts on PWB and work

When it comes to coming to work, risking disease, and needing to pay bills, people in the service business must make complicated options.^17^ This decision may be particularly challenging for those at high risk or who live with high‐risk folks. In addition, these difficulties are amplified in particular populations. Worse of all, these serve as stressors that affect a sound mind. In the long run, it can reduce productivity. The COVID‐19 epidemic has contributed to increasing professional ambiguity and lower ambivalence tolerance in career decisions, which refers to how a person perceives and reacts to gray areas.^18^ Several studies imply that workplace environments have worsened due to the COVID‐19 crisis and laborers are more prone to develop psychological issues such as stress, sadness, and anxiety.^19^, ^20^, ^21^, ^22^

## 2 METHOD

This article took the format of a literature review using existing academic work from source documents. There are three primary data collection sources: Web of Science, Scopus, and PubMed; other sources include Google Scholar dimension. However, the Scopus data set has been used for this study. Predominantly the work paid attention to English journals. The population of this study was open as it has not limited to any geographical scope. This allowed access to relevant documents related to the present study area of PWB from 1978 to 2022. The Bibliometrix R package is used for robust analysis of retrieved data.

### 2.1 About bibliometrix R package

An R‐tool (R version 4.2.1) is a complete scientific mapping study package that makes use of a library biblioshiny in the latest version of bibliometrix 4.0.0. This package allows for a quantitative examination of previous studies. In bibliometrics, published works such as journal articles and the number of times those publications have been cited are subjected to quantitative analysis and statistical tracking. Data from publications and citations may now be used quantitatively to track scientific growth and development as well as the prominence of individual authors and their contributions to the conceptual and intellectual field's landscape. Bibliometrics can also be used to evaluate the performance of researchers.^23^

## 3 RESULT

### 3.1 Annual scientific production

**Figure 1:**
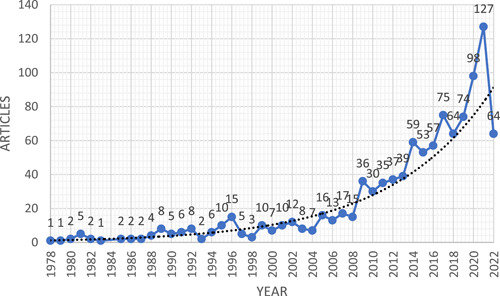
Scopus publication from 1978 to 2022 on psychological well‐being (PWB) in business and economics arenas

In response to the rapid development in business and industrialization, this topic has been the subject of a slew of academic studies. The scope of this issue will be investigated through an examination of 1053 research papers. The search term in the Scopus index was “psychological well‐being” OR “psychological wellbeing” in TITLE‐ABS‐KEY. The inclusion criteria were the papers published in the subject area in the field of Economics, Econometrics, Finance, Business, Management and Accounting only, which are in the final stage of publication, and in the English language only. Those articles published in other than the English language and articles in the press were the exclusion criteria. The annual growth rate was recorded as an exponential growth of 10.15%. Figure 1 displays the increase in research work on PWB in business and economics arenas over the course of the last few years.

### 3.2 Source dynamics

**Figure 2:**
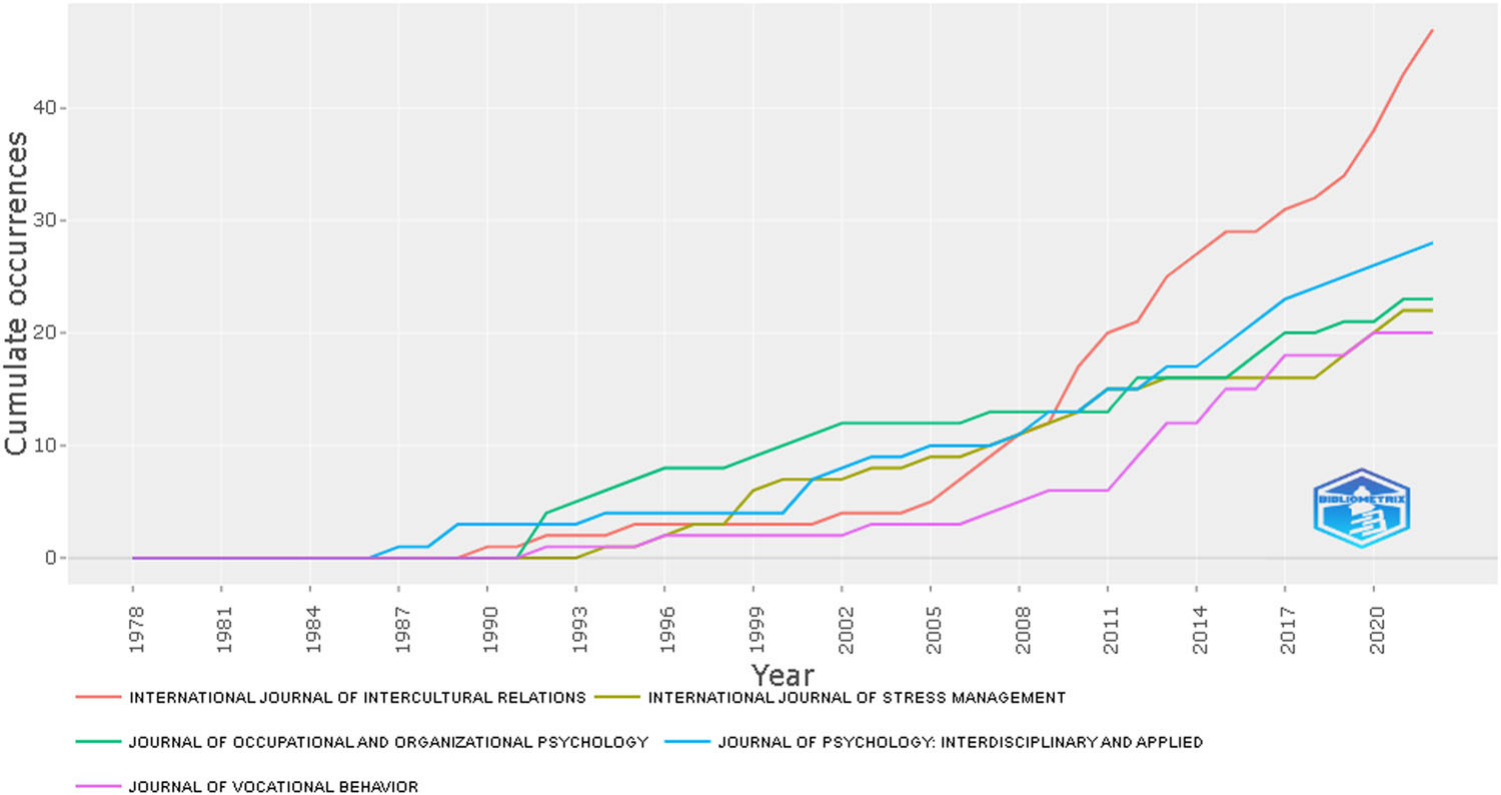
Trends in content related to the theme in various scientific publications

As shown in Figure 2, the *International Journal of Intercultural Relations* has contributed considerably to the development of this topic. It is highly specialized in this type of subject for PWB in business and economics. This well‐known open access publishes cutting‐edge research and serves as the best journal for publishing and retrieving topics related to PWB. It is also a bi‐monthly peer‐reviewed scholarly magazine that focuses on intercultural interactions.

### 3.3 Country scientific production

**Figure 3:**
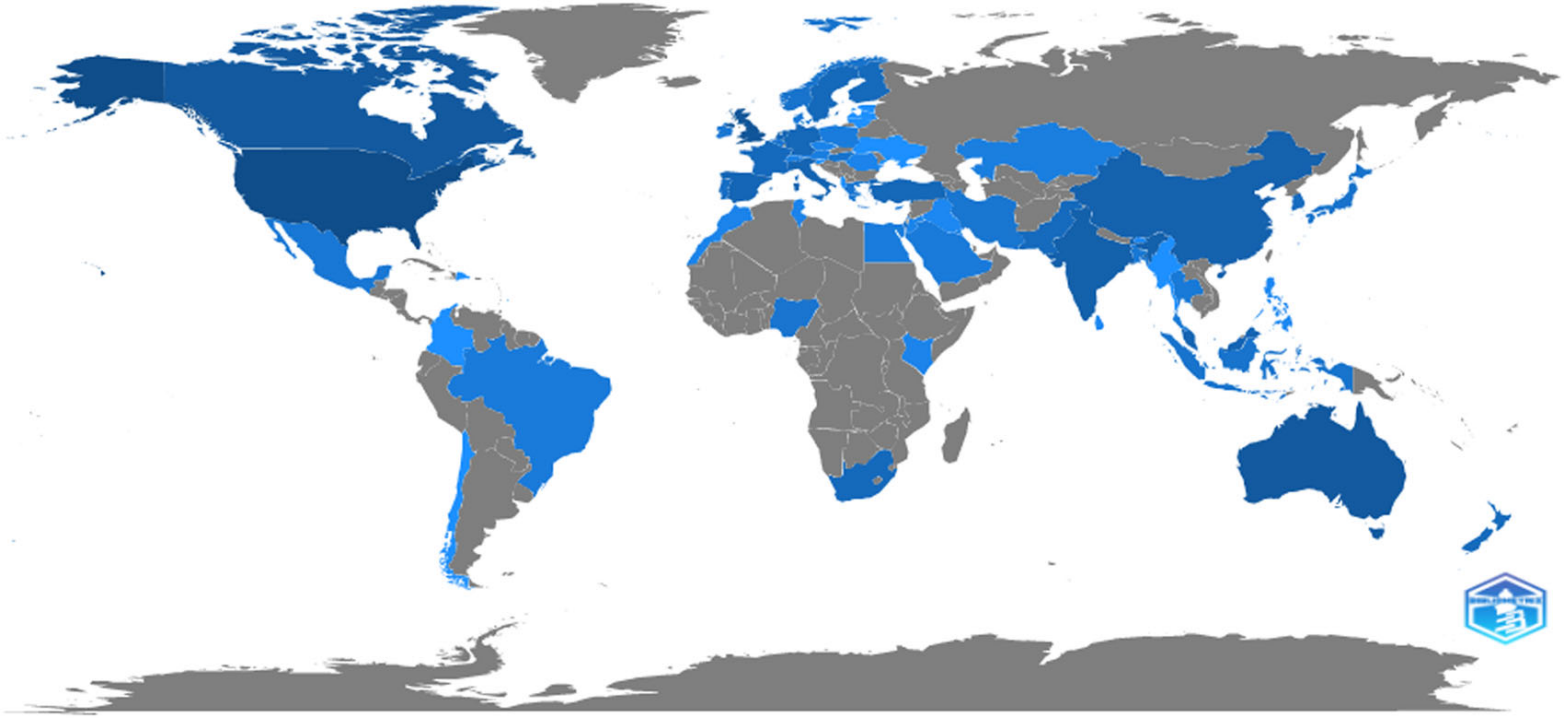
World's research development on a globe

Another essential aspect of this article is comparing the scientific output of various countries. The study reveals that academics from four different countries, like the United States (553 articles), the United Kingdom (240 articles), Australia (166 articles), and Canada (165 articles) have played a significant influence in the development of PWB in business and economics. Figure 3 depicts the world's research development on a globe. These data also indicate that most of the developing countries have not done much work within this research area (Table 1).

**Table 1:** Research conducted by the country with PWB in business and economics

Country	Frequency	Country	Frequency
USA	553	Germany	44
UK	240	Turkey	44
Australia	166	South Africa	36
Canada	165	Sweden	36
India	85	France	34
China	73	Finland	33
Netherlands	59	New Zealand	33
Italy	57	Pakistan	32
Malaysia	55	South Korea	28
Spain	50	Portugal	23

Abbreviation: PWB, psychological well‐being.

### 3.4 Country of the correspondent author

**Figure 4:**
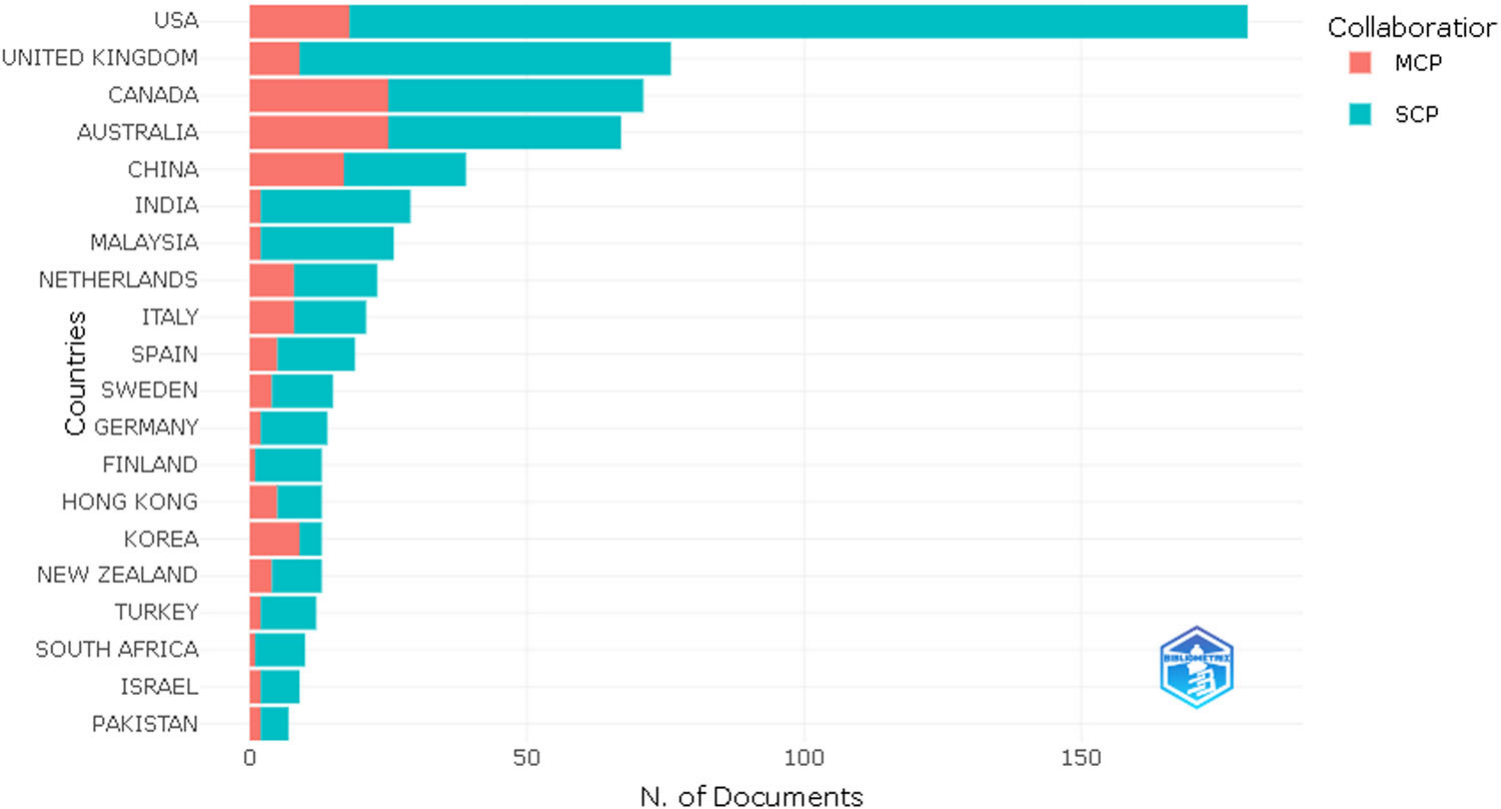
Country of the correspondent author

The corresponding nation of the corresponding author indicates whether the researchers have worked together in the same country or other countries on the same subject area. According to Figure 4, this topic has brought together researchers worldwide to collaborate on it. The data also show a fairly distributed collaboration. However, there is still scope, especially among emerging and dynamic economies. This will provide a holistic policy framework to address an all‐important topic of PWB.

### 3.5 Most cited countries

**Figure 5:**
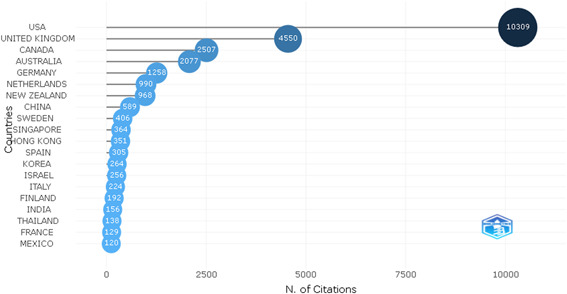
Countries with the highest number of citations

The Unites States has the most sway among countries that produce scientific research, according to a survey of the countries that have an impact on this subject. The United Kingdom, Canada, and Australia are the next most influential countries. The findings demonstrate that the leading countries account for more than 80% of the references (Figure 5).

### 3.6 Temporal analysis of the most frequent keywords

**Figure 6:**
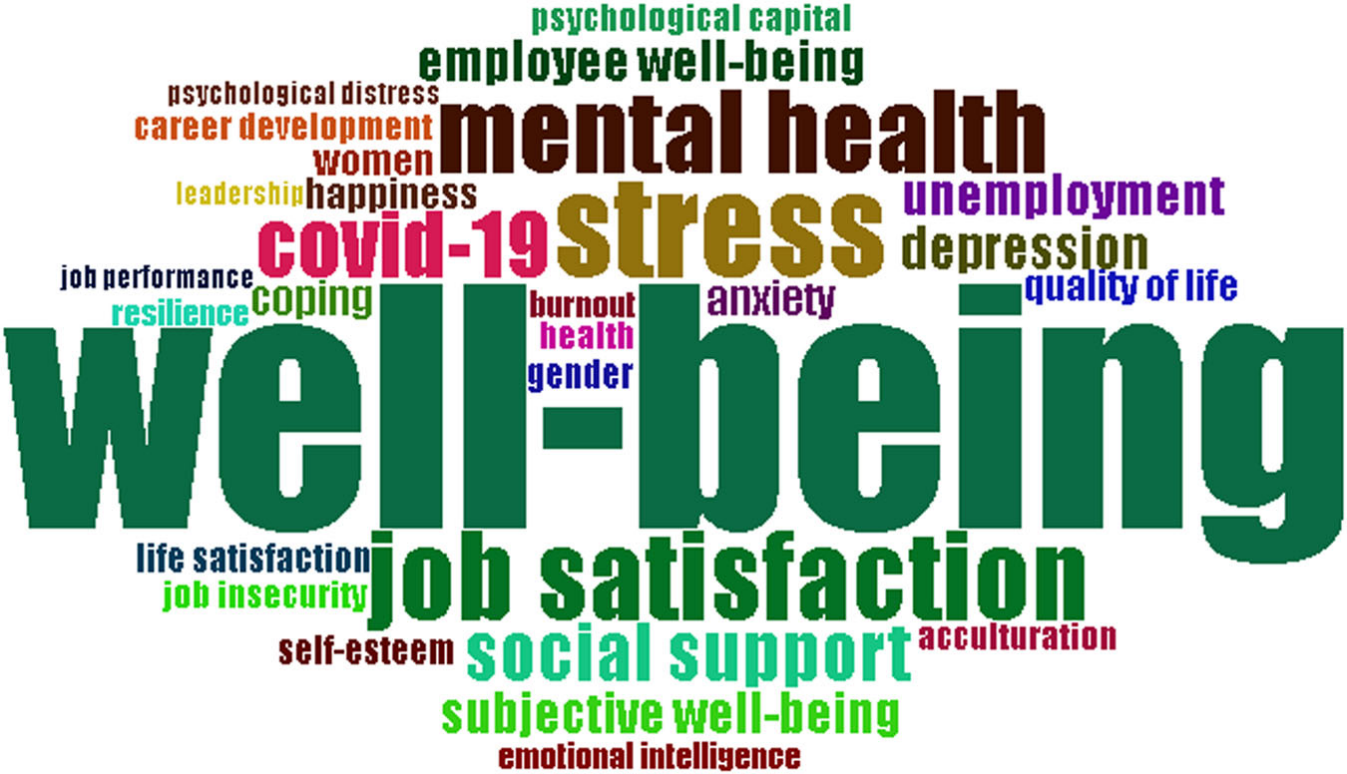
Keyword usage in a variety of research papers

In Table 2, a list of some of the keywords that have been found to appear most frequently in research done on the topic of PWB dealing with business and economics. For example, the terms “Psychological Well‐Being,” “Well‐Being,” “Stress”, and “Job Satisfaction” are all highly relevant keywords that have been identified across the sphere of the research works. In addition, Figure 6 provides an overview of the top words in terms of frequency of occurrence over a given time span. Following are some of the research hotspots identified which include the terms “Mental Health,” “COVID‐19,” “Social Support,” “Depression,” “Unemployment,” “Anxiety,” and “Coping.” All these are all associated with a higher prevalence of terms used in the content of various works.

**Table 2:** The most common keywords used in PWB research in business and economics arena

Words	Occurrences	Words	Occurrences
Psychological Well‐Being	278	Unemployment	19
Well‐Being	114	Anxiety	16
Stress	48	Coping	16
Job Satisfaction	42	Happiness	15
Mental Health	39	Quality Of Life	15
COVID‐19	32	Women	15
Social Support	28	Gender	14
Depression	20	Life Satisfaction	14
Employee Well‐Being	19	Self‐Esteem	14
Subjective Well‐Being	19	Acculturation	13

Abbreviation: PWB, psychological well‐being.

**Figure 7:**
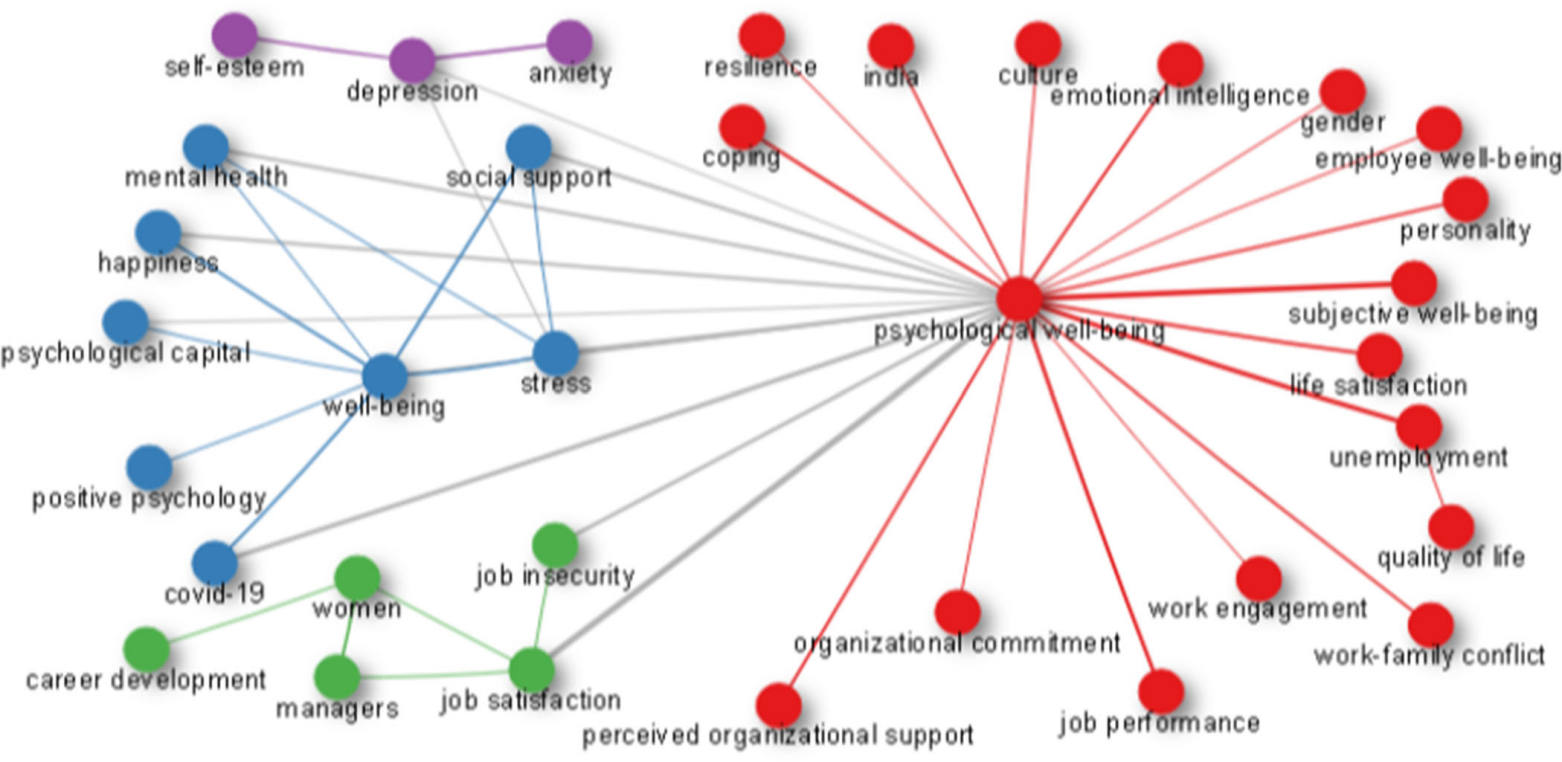
The network of co‐occurrence (1978−2022)

Through the study of the relationships between terms in the literature, a keywords co‐occurrence network (KCN) can be used to better comprehend components and the structure of knowledge within a scientific field of study. As depicted in Figure 7, on the basis of KCNs, a variety of empirical and theoretical methods have been used to analyze the relationship between study themes and their study contexts. Putting keywords together in a cluster increases the likelihood that the keywords will reflect the same subject matter. Subject keywords are distributed differently among the clusters. This illustrates the key clusters of this research, which include resilience, culture, work engagement, job insecurity, psychological capital and happiness among others.

### 3.7 The conceptual framework and interrelationships network

### 3.8 Analysis of correspondence and map of the conceptual structure

**Figure 8:**
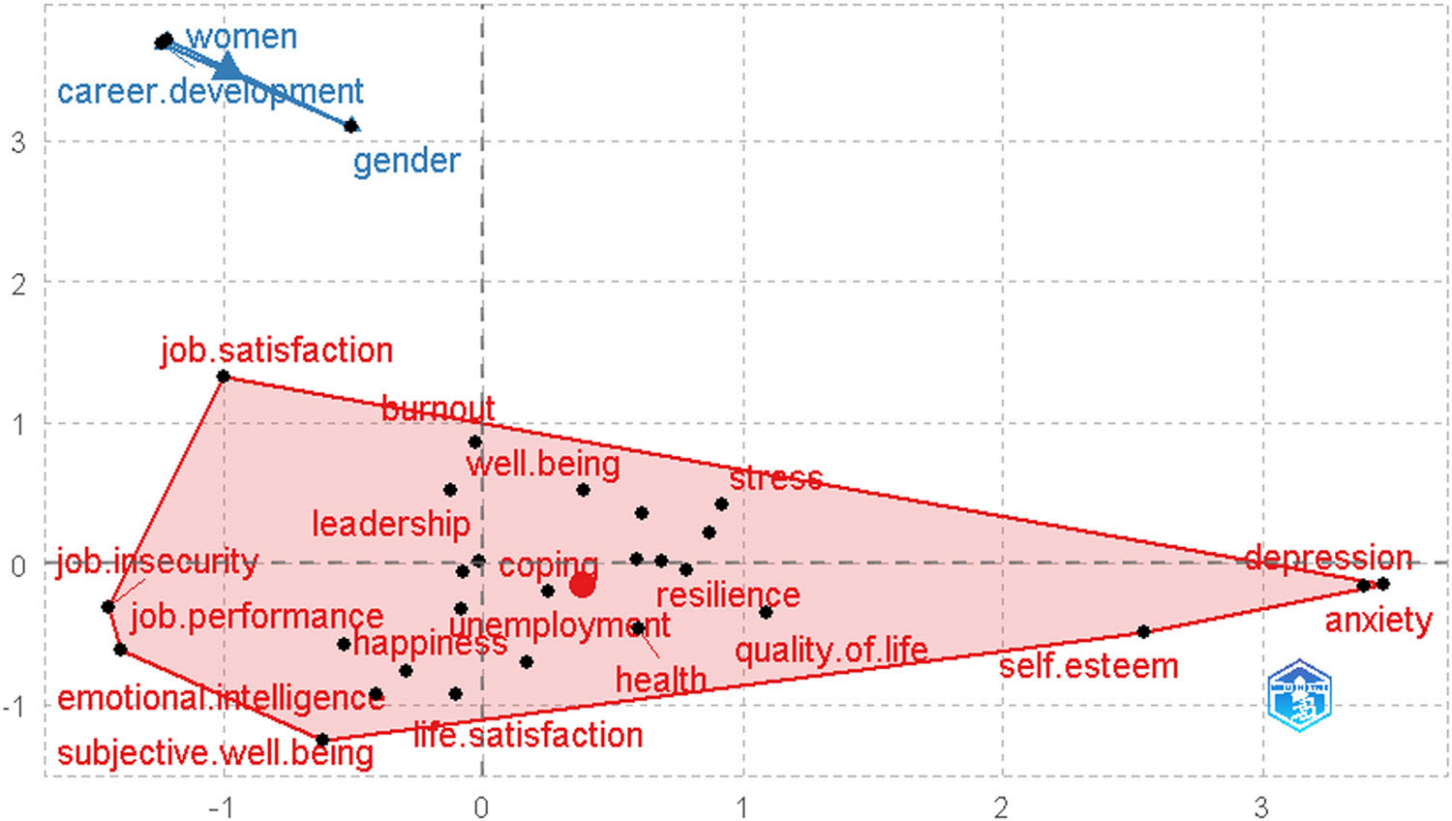
The network of co‐occurrence (1978−2022)

The coword analysis aims to portray the conceptual structure of a framework by employing words that occur in close proximity to one another. The terms, keywords plus, and authors keywords taken from abstracts or titles can all be substituted for the words in the sentence. Moreover, the map of the conceptual structure and factor of the papers with the most significant impact and the most cited documents are all produced by the conceptual structure function. Figure 8 depicts a conceptual structure map which shows that the lower cluster has the most terms indicating that the researchers' attention has been drawn to this study's topic under investigation.

### 3.9 Thematic map

**Figure 9:**
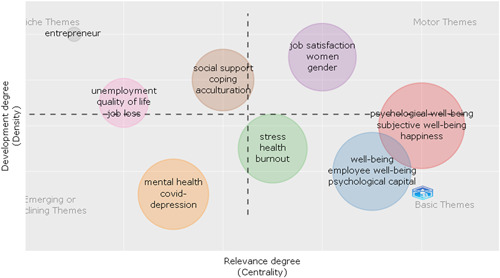
Thematic map

The formation of keyword clusters is accomplished by utilizing coword analysis, which are then regarded as themed. Figure 9 of the schematic diagram shows the density on the vertical axis. This is the degree to which a cluster's themes are linked to each other. The centrality is represented by the lateral vertical axis, which shows how many other ideas are linked to the notion. The density and centrality of a cluster are represented by a theme. Thematic maps are very intuitive plots that allow examining topics in relation to the section in which they are located. For example, the quadrant of upper‐right depicted for motor themes contains PWB, job satisfaction, women, and gender topics. The lower‐right quadrant is devoted to fundamental issues, the most important of which are stress, health, burnout, PWB, subjective well‐being, happiness, and employee well‐being, as well as psychological capital as the primary topics. The lower‐left quadrant contains emerging or vanishing themes, and subjects related to mental health, COVID, and depression can be found there. Finally, in the upper‐left quadrant, very specialized or niche issues such as entrepreneurship, unemployment, job loss, social support, coping, and acculturation can be seen (Table 3).

**Table 3:** Collaboration of countries on PWB studies in business and economics

From	To	Frequency
USA	Canada	16
USA	Korea	13
USA	Australia	11
USA	China	11
USA	United Kingdom	11
Canada	Turkey	9
United Kingdom	Australia	9
Canada	France	6
China	Hong Kong	6
United Kingdom	Italy	6
Canada	China	5
Netherlands	Germany	5
USA	Germany	5
USA	South Africa	5
Canada	Egypt	4
Canada	Japan	4
Canada	Spain	4
United Kingdom	China	4
Australia	New Zealand	3
Australia	Sweden	3
Canada	Germany	3
Netherlands	Italy	3
Netherlands	Spain	3
Spain	Portugal	3
United Kingdom	France	3
United Kingdom	Hong Kong	3
United Kingdom	Spain	3
USA	Hong Kong	3
USA	Israel	3
USA	Singapore	3
USA	Turkey	3

### 3.10 Contributions of countries to the societal structure

**Figure 10:**
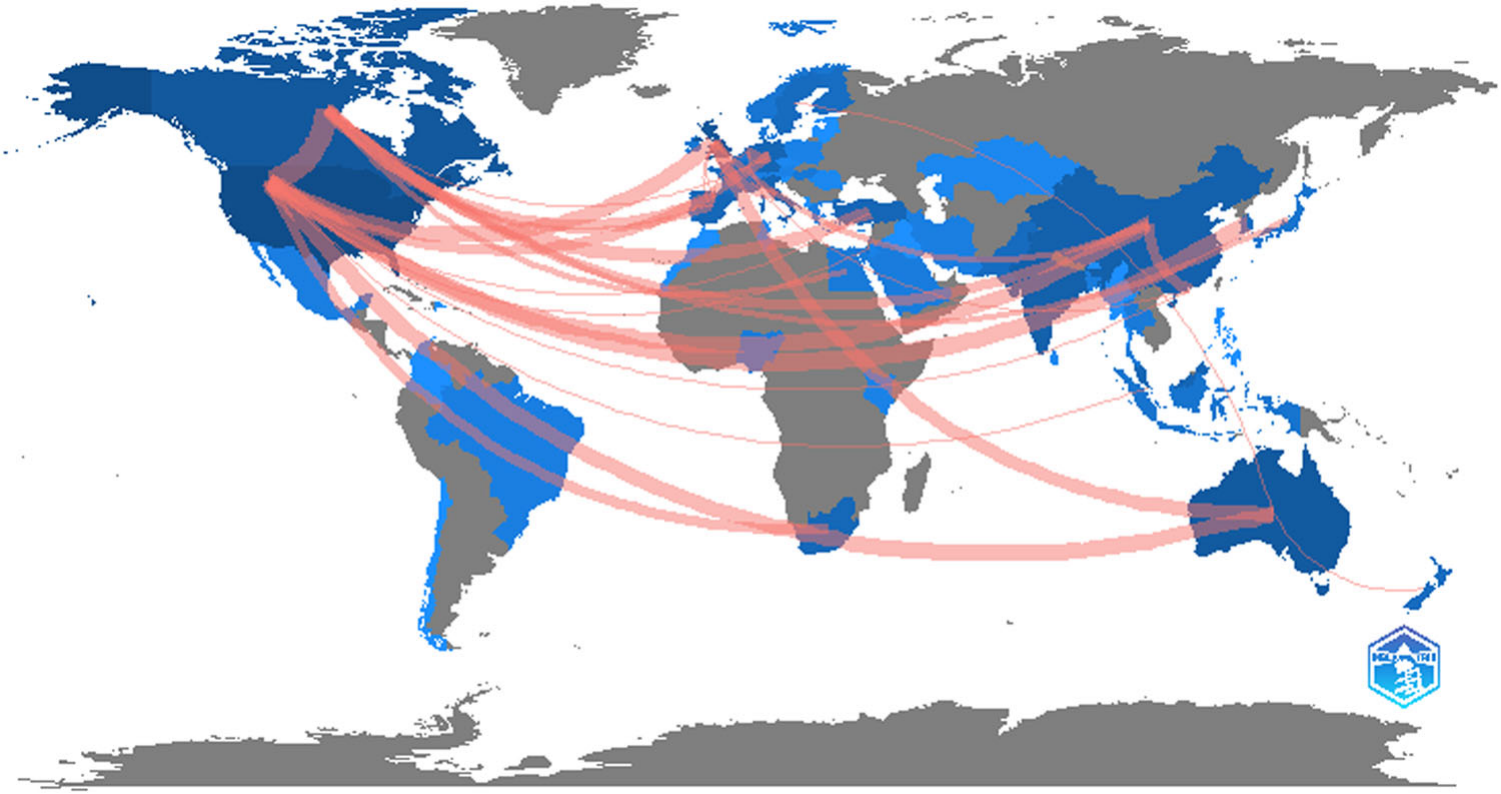
Contributions of countries

There was a lot of international cooperation to do scientific research on this subject as depicted in Figure 10.

### 3.11 Highly cited papers

As an indicator of paper influence, citations are used to assess article impact, but not exclusively. It is because the newer article has received few citations because few people have read it. The number of citations for an article does rise over time. Table 4 summarizes the cited articles. Most citations have gone to the work of Warr et al.^89^ This research compares and contrasts two studies of male manual laborers. Although based on previous work, the scales utilized solve conceptual and practical shortcomings. Participants may assess their level of concern and work involvement. Cluster‐based components of work and life happiness are also discovered. The scales' internal reliability and factoriality are established.

**Table 4:** The most cited articles

Paper	Total citations	TC per year
J Occup Organ Psychol^24^	1376	31.2727
Int J Intercult Relat^25^	777	23.5455
Rev Econ Stat^26^	744	37.2
Hum Resour Manage Rev^27^	724	34.4762
Hum Resour Dev Q^28^	707	58.9167
J Manage Psychol^29^	684	38
J Organ Behav^30^	579	28.95
J Manage^31^	578	96.3333
J Vocat Behav^32^	485	17.963
J Organ Behav^33^	409	18.5909
Acad Manage J^34^	360	17.1429
Organ Sci^35^	328	25.2308
J MARK‐A^36^	308	11.4074
Eval Program Plann^37^	294	7.1707
Eur J Work Organ Psychol^38^	287	16.8824
J Manage^39^	285	17.8125
J Occup Organ Psychol^40^	276	12.5455
Q J Econ^41^	251	35.8571
Int J Inf Manage^42^	249	49.8
J Econ Psychol^43^	231	12.8333
J Bus Ethics^44^	229	20.8182
J Manage^45^	222	8.2222
Int J Intercult Relat^46^	221	17
J Manage^47^	215	21.5
Organ Behav Hum Decis Processes^48^	211	9.5909
J Vocat Behav^49^	203	20.3
Organ Dyn^50^	199	10.4737
J Occup Organ Psychol^51^	198	7.0714
Int J Intercult Relat^52^	198	6.3871
Int J Stress Manage^53^	184	9.2
J Vocat Behav^54^	178	12.7143
Hum Relat^55^	174	6
J Small Bus Manage^56^	166	7.2174
J R Stat Soc Ser A Stat Soc^57^	166	9.2222
J Occup Organ Psychol^58^	151	3.5116
J Bus Venturing^59^	149	10.6429
J Occup Organ Psychol^60^	146	6.9524
J Vocat Behav^61^	136	45.3333
Int J Intercult Relat^62^	134	11.1667
Eur J Work Organ Psychol^63^	134	8.375
Leadersh Organ Dev J^64^	133	10.2308
J Consum Psychol^65^	132	10.1538
Uy M, 2013, J Bus Venturing	132	13.2
J Vocat Behav^66^	126	11.4545
Int J Hum Resour Manage^67^	122	6.4211
J Organ Behav^68^	122	15.25
Leadersh Q^69^	121	13.4444
J Bus Psychol^70^	121	11
J Vocat Behav^71^	120	7.5
Eur J Work Organ Psychol^72^	119	6.6111
J Leadersh Organ Stud^73^	117	11.7
Int J Stress Manage^74^	117	5.087
J R Stat Soc Ser A Stat Soc^75^	115	6.7647
J Travel Res^76^	114	4.56
Int J Tour Res^77^	113	7.5333
J Psychol Interdiscip Appl^78^	112	9.3333
Int J Intercult Relat^79^	110	8.4615
Hum Resour Manage Rev^80^	108	9
J Public Econ^81^	108	7.2
J Constr Eng Manage^82^	108	8.3077
J Ecotour^83^	107	5.9444
Int J Stress Manage^84^	106	5.8889
J Organ Behav^85^	103	4.2917
Int J Intercult Relat^86^	101	6.3125
J Occup Organ Psychol^87^	100	3.3333
Group Organ Manage^88^	100	5

Abbreviation: TC, total citation.

The study of Searle and Ward^90^ is the second most cited paper. In cross‐cultural transitions, they believe there are psychological and social adjustment forms. In this study, 155 students from Malaysia and Singapore who were studied in New Zealand asked for details concerning their mental health and sociocultural competence concerning expected difficulty, cultural distance, quantity and quality of social interactions with both hosts and fellow nationals, attitudes toward hosts, extraversion, life changes, and personal factors like age, sex, and length. All of these factors contributed to a 34% variation in psychological adjustment. Due to cultural distance, tough expectations, and unhappiness, social adjustment differs by 36%. While psychological and societal adjustment are linked, it must be thought of independently. This gave rise to a new paradigm within which PWB might be conceived.

The third highly cited work is by Tella et al.^91^ They demonstrate how macroeconomic changes have an impact on national happiness. PWB shows strong microeconomic tendencies among a quarter million Europeans and Americans who were randomly chosen from 1970 to 1990. Every country has the same equations for happiness. Second, fluctuations in perceived happiness are related to macroeconomic indicators such as gross domestic product (GDP). The same result is obtained when personal qualities, country‐fixed effects, and year dummies are controlled for. Third, the article claims that recessions result in psychological harm, a drop in GDP, and an increase in unemployment. Democratic nations seem to offer a counterbalance. Higher unemployment benefits the advanced country's well‐being.

## 4 DISCUSSION

This study focused though important but has seen less dynamic research into it especially within the space of business. Additionally, the COVID‐19 incident has made this essential subject relevant in today's world. Despite the fact that the PWB has significance in several fields of research, it indicates that the previous 10 years (starting in 2010) have seen a significant increase. However, there has been a slowdown in academic research related to PWB since last year (2021). Due to two big international disruptions—COVID‐19 and the Russia−Ukraine conflict—it is expected more work will be focused on PWB. The scholarly input has been from industrial countries. However, the trends show a corresponding good relationship between governments and institutions. However, it will be encouraging to see more work from developing countries which are often hard hit by international disruptions. Relevant articles on the subject have appeared in publications with a high impact factor. This shows the importance the academic world puts on literature evolving around PWB.

### 4.1 PWB: Future research opportunities

Work can provide a lot of personal satisfaction, creativity, and meaning. It can be entertaining, unique, and expressive. The same work can also be a reason for someone to lose touch with reality and be stressed. Many work personnel aspire to leave their mark on the world. They are described as visionaries passionate about their work and feel a deep innate connection with the products and services they contribute. As a result, the very process of self‐actualization through purposeful, authentic, and self‐organized activities that can lead to a fulfilling and fully functioning life is embodied by the nature of entrepreneurial work. Moreover, the subjects linked to PWB in business and economics arena were quite broad and related to a variety of macro (associated with communities or nations) and micro (individual) elements of health, psychology, economics, society, and culture. Therefore, varied work will need to be done to streamline them and provide further policy framework to deal with PWB.

### 4.2 Areas worth exploring in other studies

#### 4.2.1 Well‐being is an essential dependent variable in business

This study provides a framework to make well‐being a frontline variable in deciding the output of most businesses. Sánchez‐García et al.^92^ asserted that there is a rising tendency in the business world and that a sizable portion of people has plans to begin some kind of activity soon. However, it has maintained that PWB serves as an engine in the lives of the entrepreneur and its employees since it is essential for business owners to be in good states of mind and that the same is required of their staff if they are to sustain their enterprises over time. These advantages are beneficial for both the expansion of the company and the well‐being of the workforce. But ultimately, everything revolves around the human desire to achieve the greatest possible level of well‐being. Exploratory work can also be done in this tangent to quantify its contribution to the business and economic area. PWB is not just an essential dependent variable but also a key predictor of many desirable results.^93^ People who are happy, for example, have more fulfilling jobs.^94^

#### 4.2.2 Institutional influences on well‐being

Until now, the majority of study has concentrated on positive feelings. Emotions are crucial in business because entrepreneurs and workers alike must make judgments in the face of uncertainty, under time constraints, and with few historical precedents to help them. Therefore, studies on how negative PWB can influence all the above action is worth exploring to understand how best to manage it. Ignoring the activation components of emotions is a fundamental flaw in this set of investigations. The introduction of COVID‐19, for example, acted as a stressor in the corporate environment. The battle with Russia has also added to the market's unpredictability. These are not far‐fetched components of emotion that can impact a worker's phycology, and very minimal work has been done to access such components to guide it. Major among them are some working environments or working conditions. Overall, research into well‐being as a resource or trigger of an active working environment is relatively limited. Further studies are expected to add to more understanding of well‐being as an essential psychological resource and mechanism for improving company prospects.

#### 4.2.3 Changing the phase of technology on PWB

More workers are worried about the new phase of technology taking over their work. Many are also suffocated by the changing phase of new technology and how quickly they are expected to adopt it. How the adoption of technology advancements affects employee PWB, such as employee anxiety and satisfaction, is a crucial yet understudied study issue. According to recent research, technological innovation may significantly impact both job design and employee performance.^95^ Artificial intelligence technology, advanced analytics, digitalization, and automation are all posing challenges to workers' workflows, which can be irritating for some. Despite the increased scholarly interest in new technologies, there are certain information gaps in the literature, particularly in how it impacts users and their interactions with technology. However, in the age of technology, a sole emphasis on personnel management is inadequate for enterprises to safeguard workers' PWB, given modifications processes.^96^ However, previous research has not looked at the circumstances leading to technology innovation driving SME employee well‐being. Given the velocity of technological transitions in organizations and the changing business environment, this absence is seen as a crucial knowledge gap in the literature.^97^

### 4.3 Implications

Understanding how the COVID‐19 issue has impacted PWB is an area of research that should be pursued. Another element to consider is the impact of such dynamics on productivity and corporate performance. Furthermore, crises have affected the education of prospective employees who are presently enrolled in school. More research is needed to identify the career‐related demands that these various groups may have. Specific COVID‐19 factors should be investigated using well‐established career development theories to inform the creation of career development interventions. Inequalities in the race and socioeconomic class, as well as generational and age differences, should all be taken into account. Because the COVID‐19 pandemic has affected both, studies addressing the links between professional success and mental health are essential. This will allow you to get ahead of the problem, as physiological impairment is a disorder that does not resolve quickly but develops over time.

The COVID‐19 disaster has drastically altered people's lives and livelihoods. While the majority of research has focused on the current negative repercussions, beneficial changes in everyday life have gotten less attention. Other hypotheses focus on the opportunity it provides for most workers to get the needed rest and heal from years of hard labor without having to give up vacations and other benefits. Some feel it also aids PWB by including the perspective of spending time with family and bonding. However, most of this has been speculative, and more research is needed to comprehend these phenomena fully.

### 4.4 Future studies

The bibliometric technique in conjunction with content analysis assisted in identifying numerous critical research needs. Specifically, the previous work of this research area lies within the health debate but losing stride within the business force which calls for more related work. Based on the current trajectories, more work is needed to fill this gap, especially from developing countries that seem to be doing little to understand the inputs of PWB on the business and economic environment. Also, there was not much conceptualization within this study framework. Hence, it has been proposed that future studies be done in this area to understand how existing models and theories are applied to meet the need of this millennium in which several stressors have compounded.

## 5 CONCLUSION

This research reveals that the studies on PWB have been growing in notoriety within the last four decades, with widespread recognition in the commercial world. The rise in stressors can affect the performance of workers and their delivery of production. The results are grounds for concern as an increase in PWB work was expected, but data depicted the opposite. The data also show a fairly distributed collaboration. However, there is still room to be done, especially among emerging and industrious economies. This will provide a holistic policy framework to address an all‐important topic of PWB. It is likely to consider the prospect in decline in this research area because of the subcategory review and the keywords' lack of the term business. As the wide range of keywords and their low density demonstrate, the subject matter at hand is extremely diverse. Because of this, it needs to be addressed in greater detail. This issue can be further investigated from three perspectives: PWB, stress, and work satisfaction in the industrial environment.

## AUTHOR CONTRIBUTIONS

**Satish Ambhore**: Conceptualization; data curation; formal analysis; resources; software. **Elvis Kwame Ofori**: Formal analysis; investigation; resources; writing – review & editing. All authors have read and approved the final version of the manuscript [CORRESPONDING AUTHOR or MANUSCRIPT GUARANTOR] had full access to all of the data in this study and takes complete responsibility for the integrity of the data and the accuracy of the data analysis.

## CONFLICT OF INTEREST

The authors declare no conflict of interest.

## TRANSPARENCY STATEMENT

The lead author Elvis Kwame Ofori affirms that this manuscript is an honest, accurate, and transparent account of the study being reported; that no important aspects of the study have been omitted; and that any discrepancies from the study as planned (and, if relevant, registered) have been explained.

## Data Availability

The data sets used and/or analyzed during the current study are available from the corresponding author on reasonable request.
